# Transcriptomics Profiling Identifies Cisplatin-Inducible Death Receptor 5 Antisense Long Non-coding RNA as a Modulator of Proliferation and Metastasis in HeLa Cells

**DOI:** 10.3389/fcell.2021.688855

**Published:** 2021-08-23

**Authors:** Dilek Cansu Gurer, İpek Erdogan, Ulvi Ahmadov, Merve Basol, Osama Sweef, Gulcin Cakan-Akdogan, Bünyamin Akgül

**Affiliations:** ^1^Noncoding RNA Laboratory, Department of Molecular Biology and Genetics, Ízmir, Institute of Technology, Izmir, Turkey; ^2^Izmir Biomedicine and Genome Center, Ízmir, Turkey; ^3^Izmir International Biomedicine and Genome Institute, Dokuz Eylul University, Ízmir, Turkey

**Keywords:** cisplatin, apoptosis, lncRNAs, proliferation, metastasis, transcriptomics

## Abstract

Cisplatin is a well-known cancer chemotherapeutic agent but how extensively long non-coding RNA (lncRNA) expression is modulated by cisplatin is unknown. It is imperative to employ a comprehensive approach to obtain a better account of cisplatin-mediated changes in the expression of lncRNAs. In this study, we used a transcriptomics approach to profile lncRNAs in cisplatin-treated HeLa cells, which resulted in identification of 10,214 differentially expressed lncRNAs, of which 2,500 were antisense lncRNAs. For functional analyses, we knocked down one of the cisplatin inducible lncRNAs, death receptor 5 antisense (DR5-AS) lncRNA, which resulted in a morphological change in HeLa cell shape without inducing any cell death. A second round of transcriptomics-based profiling revealed differential expression of genes associated with immune system, motility and cell cycle in DR5-AS knockdown HeLa cells. Cellular analyses showed that DR5-AS reduced cell proliferation and caused a cell cycle arrest at S and G2/M phases. Moreover, DR5-AS knockdown reduced the invasive capacity of HeLa cells in zebrafish xenograft model. These results suggest that cisplatin-mediated pleiotropic effects, such as reduction in cell proliferation, metastasis and cell cycle arrest, may be mediated by lncRNAs.

## Introduction

Cisplatin, a universal chemotherapeutic drug, is used in the treatment of a diverse array of cancer (Kelland, 2007). As an alkylating-like agent, the platinum atom of cisplatin interacts with purines in DNA and induces crosslinks that lead to DNA damage and cell cycle arrest (Siddik, 2003). Such cellular perturbations trigger numerous signal transduction pathways and inflammatory pathways that trigger apoptosis. This DNA-damage-induced cell death is exploited in combination chemotherapies due to its synergistic effect. However, many patients develop resistance to chemotherapeutic drugs, including cisplatin (Köberle et al., 2010). Thus, it is important to unravel the molecular mechanisms underlying the mode of action of platinum-based chemotherapeutic drugs.

Long non-coding RNAs (lncRNAs), non-protein-coding transcripts longer than 200 nt in length, are novel regulators of both transcriptional and post-transcriptional gene expression involved in numerous cellular processes such as growth, cell death, and differentiation (Geisler and Coller, 2013; Uszczynska-Ratajczak et al., 2018). Transcribed largely by RNA polymerase II and III, several different biotypes of lncRNAs exist in the cell and they regulate gene expression by interacting with various macromolecules including DNA, RNA or proteins (Kopp and Mendell, 2018). The existing studies suggest that lncRNAs have the potential to modulate the molecular effect of cisplatin (Hu et al., 2018). Accordingly, bioinformatics analyses and microarray-based profiling of lncRNAs in different cancer cell lines and tissues treated with cisplatin clearly indicate that lncRNAs are involved in cisplatin-mediated cellular processes (Yang et al., 2013; Hu et al., 2017; Zhu et al., 2020). Additionally, lncRNAs have been linked to cisplatin resistance through various molecular mechanisms that involve modulation of transcription (Fan et al., 2020), miRNA activity (Cheng et al., 2019; Longqiu et al., 2020), post-translational modification (Wang et al., 2018), or epigenetic silencing (Yu et al., 2019).

Despite the existence of several studies that associate lncRNAs with cisplatin’s mode of action, a robust profiling study is needed to obtain a comprehensive analysis of all types of lncRNAs. Here, we present the lncRNA profile of HeLa cells treated with cisplatin. Our data show that cisplatin induces a broad repertoire of lncRNAs including lincRNAs, antisense lncRNAs and intronic lncRNAs. Death receptor 5 antisense (DR5-AS) lncRNA, a cisplatin-inducible natural antisense transcript (NAT) that is antisense to the DR5 receptor, modulates cell fate as its knockdown changes HeLa cell morphology. Transcriptomics analysis of DR5-AS-knockdown cells has revealed that DR5-AS modulates cell cycle, proliferation and metastasis without affecting cell death.

## Materials and Methods

### Cell Culture and Drug Treatments

HeLa cells, obtained from DKFZ GmbH (Germany), were cultured in RPMI 1640 (with 2 mM L-Glutamine, Gibco, United States) supplemented with 10% fetal bovine serum (FBS) (Gibco, United States) in a humidified atmosphere of 5% CO_2_ at 37°C. After optimization of dose and time kinetics for apoptosis rate, cisplatin (Santa Cruz Biotechnology, United States) treatments were carried out in triplicates as described previously (Yaylak et al., 2019). A similar approach was followed for tumor necrosis-factor related apoptosis-inducing ligand (TRAIL) (Enzo Life Sciences, United States) treatment.

### Total RNA Isolation, RNA-Seq, and qPCR

Total RNA was isolated using TRIzol^TM^ (Life Technologies, United States) according to the manufacturer’s instructions. Nuclear and cytoplasmic RNAs were isolated using the cytoplasmic and nuclear RNA purification kit (Norgen Biotek, Canada). Trace DNA contamination was removed with the TURBO DNA-free^TM^ kit (Invitrogen, United States).

A total of 5 μg total RNAs from three biological replicates of cisplatin-treated HeLa cells were used for library preparation and run on Illumina HiSeq 2500 by FASTERIS to identify differentially expressed lncRNAs^1^. The RNA-seq data were processed by using the bioconda environment (Grüning et al., 2018) with the following tools: quality check with FastQC (Andrews et al., 2012), adapter trimming by Cutadapt (Martin, 2011), ribosomal RNA filtering by SortMeRNA (Kopylova et al., 2012), reference genome alignment via STAR-aligner (Dobin et al., 2013), counting reads with featureCounts, final quality check with MultiQC (Ewels et al., 2016), and differential gene expression analysis by DESeq2, biomaRt and pheatmap package in R (Durinck et al., 2009, 2005; Love et al., 2014). To identify differentially expressed mRNAs in DR5-AS knockdown HeLa cells, a similar RNA sequencing was conducted with total RNAs (3 replicates) isolated from control and DR5-AS knockdown cells (Fasteris SA, Switzerland). Differentially expressed mRNAs were subjected to Pathway Enrichment Analysis by Reactome database (Jassal et al., 2020) and visualized with ggplot2 package in R platform (Wickham, 2006). Both RNA-seq data were deposited into the Gene Expression Omnibus under the accession numbers GSE160227 and GSE165560.

For qPCR analyses, cDNA was prepared using RevertAid first strand cDNA synthesis kit according to manufacturer’s instructions (Thermo Fisher Scientific, United States). qPCR reactions were prepared with GoTaq^®^ qPCR Master Mix (Promega) and RT^2^lncRNA qPCR assays for DR5-AS (Qiagen Cat., LPH15855A-200), CAMTA-AS (Qiagen Cat., LPH13091A-200), and FAF1-AS (Qiagen Cat., LPH05521A-200). Other qPCR primer sequences are presented in Table 1. GAPDH was used for normalization.

**TABLE 1:** Primer sequences employed throughout the study.

Gene name	Forward 5′-3′	Reverse 5′-3′
*PTPRU*	ACCACCTACCTGTTCTCCGT	CACCTGGTACACACTGATGGG
*VAV3*	AAGAGATAATGAGACCCTTCGTGA	AGGTAGAAGCCATAAGACCACTTT
*IL1R1*	TCTTCTCTGGAGGCTGATAAATGC	ACACAAGTCCTCCGTCTCCT
*C5*	TCTGCGTATGCTCTTTCCCTG	GCATTGATTGTGTCCTGGGTT
*SPINT2*	TATGGGGGCTGTGACGGAAA	GAACCACCACCTTTGAGCCA
*DR5*	CAGGTGTGATTCAGGTGAAGTGG	CCCCACTGTGCTTTGTACCTG
*ANAPC4*	ATAGACTCTTGGTCCAGCTGCC	TGCATGGTACGGGTGGGAATAG
*ANAPC2*	CAGTGACGACGAGAGCGACT	AGGCCCAGTCACCACAAACA
*HMGA2*	ATAAGCAAGAGTGGGCGGGT	TGAATGCCCGACGTCACAAG
*CENPP*	CATCCTGCAGACAGGGAGACAG	CTGTGTGACCTGGAGCTGATCTT
*JUN*	CTGTTGACAGCGGCGGAAAG	CACTTGTCTCCGGTCCTCCC
*GADD45B*	CGACATCAACATCGTGCGGG	AGACAATGCAGGTCTCGGGC
*P21*	CTGTGATGCGCTAATGGCGG	CCTCCAGTGGTGTCTCGGTG
*NFKBIA*	CGGAGTTCACAGAGGACGAG	CCCTTTGCGCTCATAACGTC
*IL8*	CTGTCTGGACCCCAAGGAAAA	TGAATTCTCAGCCCTCTTCAAAAAC
*BIRC3*	TCCTCCTTTGAGTTAGGTCTTGT	TGTCAAGTGTTTCACAGCAAAAA
*TLR4*	GCCAGGAGAACTACGTGTGA	GGAGCATTGCCCAACAGGAA
*TWIST1*	GGCCAGTTTGATCCCAGTATTTT	AAGGAAAGGCATCACTATGGACT
*SOCS3*	TGGCTTTCCTATGCTGGGTC	GGGATTCTACTCTGTGCCTCC
*PELI2*	TGAAATACGGGGAGCTGGTG	CCGCTTGTAGAGGGCAAATC
*LTBP3*	AGACTGGGCAGGGGTAGATT	GTTCTGCGACAGCGTATTGG
*COL4A4*	AGATGCCTACTGCAAGGGTG	TACCTCCTCTTTAGCCCCGT
*ITGA11*	GCAAGAAGACGTGGGAATGC	CCTCCCGGGTCACTTTTGTT
*SH3PXD2A*	GGGTGGCTGTTTTGCTGTTC	CCCTACCATTTTGCGTTGCC
*COL4A3*	ACATGACCCAGAGGACAGCA	TTGACAGCAAACACGTGAGC
*P53*	AAACTACCAACCCACCGACC	TCTGGCCTTGAAACCACCTT
*GAPDH*	ACTCCTCCACCTTTGACGC	GCTGTAGCCAAATTCGTTGTC
*DR5-AS*	Qiagen Cat. No. LPH15855A-200	
*MALAT-1*	Qiagen Cat. No. LPH18065A-200	
*FAF1-AS*	Qiagen Cat. No. LPH05521A-200	
*CAMTA1-AS*	Qiagen Cat. No. LPH13091A-200	

### Rapid Amplification of cDNA Ends (RACE) and Construction of Overexpression Vectors

RACE-cDNA was synthesized according to the manufacturer’s protocol for 5′/3′ RACE Kit 2^nd^ generation (Roche, Switzerland; Cat.No. 03353621001). First cycle forward primer for DR5-AS: 5′-GGCGTCCCATGCGTTGTCCCCTGCACAT-3′, reverse primer: 5′-GGACTCTTTCTTCCAGGCTGCTTCCCTT-3′. Second cycle forward primer: 5′-GGCCTCAAAGCCCAGAGG GAGCCAGTC-3′, reverse primer: 5′-TTTTTCTCATGTGAC TTGTCTCATG-3′, oligo d(T)-Anchor primer: 5′-GACCACGC GTATCGATGTCGACTTTTTTTTTTTTTTTT-3′, PCR anchor primer: 5′-GACCACGCGTATCGATGTCGAC-3′, Control primer for neo 1: 5′-CAGGCATCGCCATGGGTCAC-3′, neo 2: 5′-GCTGCCTCGTCCTGCAGTTC-3′, neo 3: 5′-GATTGCACGCAGGTTCTCCG-3′. The 2648-bp cDNA was cloned into the *Nhe*I-*Xho*I site in pcDNA3.1 plasmid to obtain pcDNA3.1-DR5-AS and was verified by sequencing. The empty vector was used as the negative control. Amplified plasmids were isolated using an endotoxin-free plasmid isolation kit (Macherey-Nagel, Germany).

### Cell Transfection

Cells were seeded in 6-well plates at a density of 80,000 cells/well 1 day prior to transfection and transfected with DR5-AS LNA GapmeR (Qiagen, United States) at a concentration of 40 nM or with 1500 ng of pcDNA3.1-DR5-AS with the Fugene HD transfection reagent (Promega, United States) in a 2-mL final volume. Negative GapmeR and empty vector were used as negative control groups for knockdown and overexpression, respectively. Media was changed 1 h post-transfection in overexpression experiments. Overexpression for rescue experiments was conducted 8 h post-silencing under conditions similar to individual transfections. Transfected cells were fixed with methanol for brightfield microscopy and stained with DAPI (Sigma, United States) for fluorescent microscopy (Kapuscinski, 1995). Cell morphology was analyzed with Fiji (Schindelin et al., 2012).

### Analysis of Apoptosis, Proliferation, and Cell Cycle

To determine apoptotic cells, biological replicates were trypsinized by 1× Trypsin-EDTA (Gibco, United States) and washed in 1× cold PBS (Gibco, United States), followed by resuspension in 1× Annexin binding buffer (Becton Dickinson, United States). The resuspended cells were stained with Annexin V-PE (Becton Dickinson, United States) and 7AAD (Becton Dickinson, United States) followed by incubation for 15 min in dark at room temperature and analyzed by FACSCanto^TM^ (Becton Dickinson, United States). Additionally, NucRed^TM^ Dead 647 ReadyProbes^TM^ (Invitrogen, United States) were used to assess the viability of the transfected cells through a fluorescent microscope (Zeiss Observer Z1, Germany). To this end, 2 drops of the dye were applied per mL of culture medium and cells were monitored following 30 min of incubation in dark.

WST-1 assay was employed to measure proliferation rates (Roche, Switzerland). Cells were seeded at a density of 1,000 cells per well in 96-well plates 1 day prior to transfection. Transfection was carried out when the confluency has reached approximately 70-80% in a volume of 100 μl per well. Samples were incubated in a humidified atmosphere of 5% CO_2_ at 37°C for 2 h following the addition of WST-1 and absorbance was measured at 450 nm. DMSO (Applichem, Germany) (5%) (v/v) was used as positive control while media without cells was used as blank.

For cell cycle analysis, trypsinized cell pellets were fixed with cold ethanol, permeabilized with Triton X-100 (Applichem, Germany) (0.1%) and treated with RNase A (Invitrogen, United States) (Pozarowski and Darzynkiewicz, 2004). The resulting cells were then stained with propidium iodide (PI) (Becton Dickinson, United States) for 15 min prior to analysis with FACSCanto^TM^ (Becton Dickinson, United States). The population density in each cell cycle phase was calculated by ModFit LT^TM^ software.

### Cell Preparation for the Xenograft Assay

For xenograft assays, HeLa cells were transfected with control and DR5-AS GapmeR for 24 h as described above. On the day of injection, 3 wells of cells were trypsinized, collected and suspended in 50 μl PBS containing 2 μl of DiI (Cat.V22885, Thermo Fisher Scientific) as described previously (Iscan et al., 2021). Cells were suspended in 10% FBS in 1× PBS at 30,000 cells/μl concentration, kept on ice and injected within 2 h.

### Zebrafish Handling and the Metastasis Assay

Zebrafish embryos were obtained from wild type AB strain, reared under standard conditions at the İzmir Biomedicine and Genome Center (IBG) Zebrafish Facility. At 2 dpf the chorions were removed manually or enzymatically. The embryos were anesthesized with 0.02% Tricaine and aligned on a microinjection plate. Cell suspension was loaded on a capillary microneedle and 150–200 cells were delivered into the yolk sac of each embryo. The xenografts were incubated in E3 embryo medium at a 34°C incubator. Successful xenografts were selected by formation of local tumor mass in the yolk. The larvae that already has metastatic cells due to delivery into the circulation were removed from the experiment. At 4 days after injection (dpi) xenografts in which more than five cells disseminated from yolk and invaded to the body of the fish were considered metastatic (Avci et al., 2018). The number of local and metastatic tumors were recorded for each experimental group and three randomly formed replicates (18–20 larvae in each replica) were analyzed for each group. Two independent experiments were performed at different times, and the difference of metastatic rate was analyzed with a *t*-test (*P* value < 0.05 was considered significant). Zebrafish experiments were performed according to regulations with permission from IBG Local Animal Experiments Ethics Committee with the approval number 2021-07.

## Results

### Cisplatin Induces a Plethora of Long Non-coding RNAs

**FIGURE 1:**
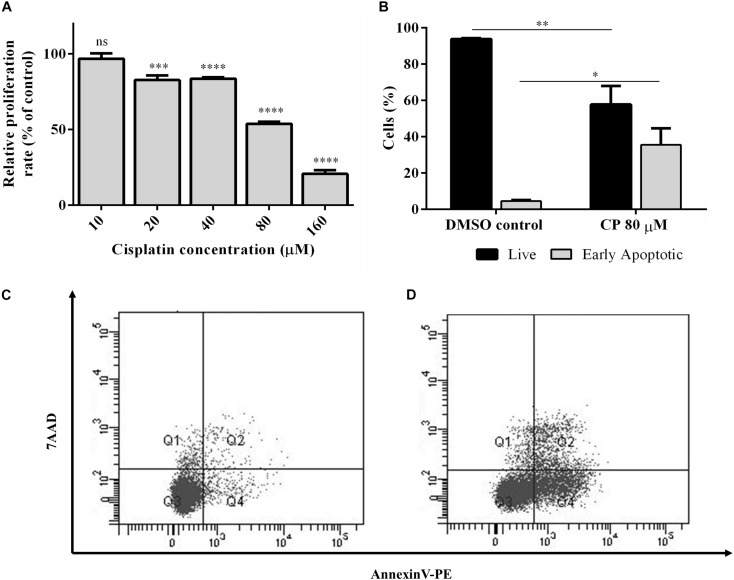
Cisplatin-induced apoptosis and proliferation in HeLa cells. **(A)** Proliferation kinetics of HeLa cells. HeLa cells were treated with a range of cisplatin concentrations for 16 h and the proliferation rate was measured by WST-1 assay. **(B)** Percentage of live and early apoptotic population of HeLa cells. Hela cells were treated with 80 μM cisplatin (CP) for 16 h and the rate of apoptotic cells were determined by Annexin-V and 7AAD staining. The stained cells were analyzed by flow cytometry. DMSO (0.1%) was used as negative control. **(C,D)** The population distributions of DMSO control and CP-treated groups as flow cytometry dot blot graphs, respectively. ns, non-significant, *p* > 0.05, ^∗^*p* ≤ 0.05, ^∗∗^*p* ≤ 0.01, ^∗∗∗^*p* ≤ 0.001, and ^****^p ≤ 0.0001.

**FIGURE 2:**
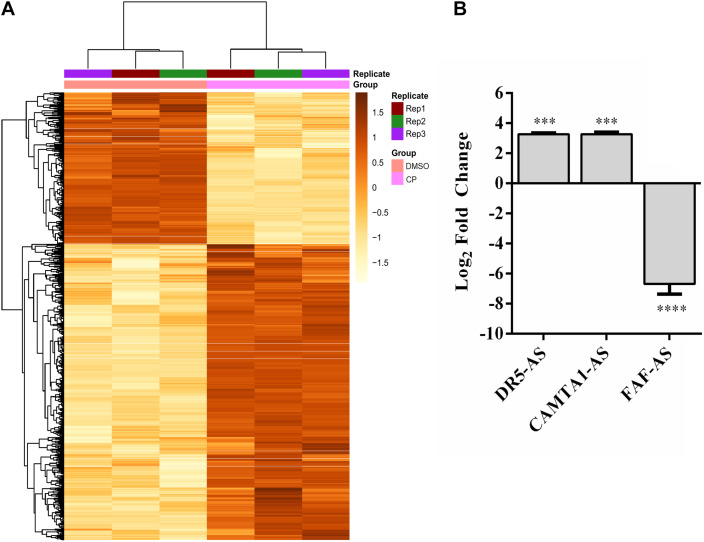
Differentially expressed lncRNAs in cisplatin-treated HeLa cells. **(A)** Heatmap of top 1,000 differentially expressed transcripts in cisplatin- and DMSO-treated HeLa cells. HeLa cells were treated with cisplatin as explained in Figure 1B. **(B)** qPCR analyses of candidate lncRNAs. qPCR was carried out with total RNAs isolated from control (0.1% DMSO) and cisplatin-treated HeLa cells. Relative expression of candidate genes was normalized against GAPDH. ^∗∗∗^*p* ≤ 0.001, ^****^p ≤ 0.0001.

Cisplatin, a chemotherapeutic drug with pleiotropic effects, is known to modulate several cellular properties. Thus, we first treated HeLa cells with varying concentrations of cisplatin to examine its effect on HeLa cells. Cisplatin at a concentration of 80 μM lowered the proliferation rate to 57.6% (Figure 1A) while inducing approximately 35.5% of apoptosis, as determined by Annexin V-positive early apoptotic cells, compared to the control DMSO (Figures 1B–D). To determine the extent of differentially expressed lncRNAs, we subjected three replicates of total RNAs isolated from control and cisplatin-treated cells to RNA sequencing. The average read was 16,518,795. The unsupervised hierarchical cluster of differentially expressed transcripts is presented in Figure 2A. Cisplatin treatment resulted in the differential expression of 10,214 lncRNAs (twofold or higher, *P* < 0.05). When we classified the differentially expressed lncRNAs based on their biotypes, we noticed that the majority of differentially expressed lncRNAs were antisense lncRNAs (2,500) and lincRNAs (1,356). However, it is quite interesting that 155 intron-derived lncRNAs were stably expressed upon cisplatin treatment. qPCR data of a select number of antisense lncRNA was congruous with the RNA-seq data (Figure 2B).

### DR5-AS Is a Cisplatin Inducible Nuclear Antisense lncRNA

**FIGURE 3:**
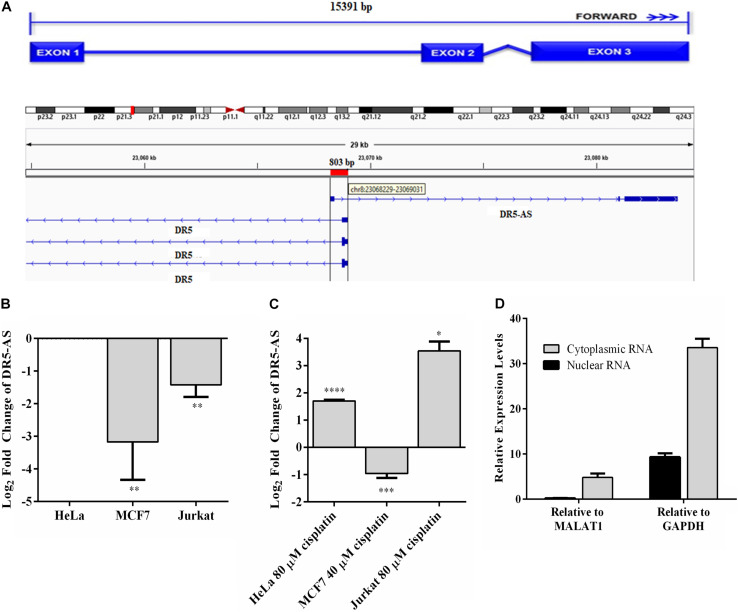
Genomic location of DR5-AS and its expression in cisplatin-treated HeLa cells. **(A)** Schematic representation of DR5-AS (ENSG00000246130) and interference with DR5 transcript variant combined with the Ensembl structure. **(B–D)** qPCR analysis of DR5-AS expression in untreated cells (**B**, normalized to DR5-AS expression in HeLa cells), cisplatin-treated cells (**C**, normalized to DR5-AS expression in DMSO-treated cells), and fractionated cells **(D)**. Cisplatin treatment and qPCR analyses were performed as explained in Figure 2B. Subcellular localization of DR5-AS was determined by qPCR analyses of nuclear and cytoplasmic RNAs isolated from cisplatin-treated HeLa cells. MALAT1 and GAPDH were used as markers for nuclear and cytoplasmic fractions, respectively. Ct values for the cytoplasmic and nuclear GAPDH were 9.7 and 13.9, respectively. Ct values for DR5-AS for the cytoplasmic and nuclear fractions were 25.2 and 20.8, respectively. *p* > 0.05, ^∗^*p* ≤ 0.05, ^∗∗^*p* ≤ 0.01, ^∗∗∗^*p* ≤ 0.001, and ^****^p ≤ 0.0001.

Of 10,214 differentially expressed lncRNAs, DR5-AS stroke our attention because (1) it is antisense to the death receptor 5 (Figure 3A, DR5); (2) it appears to be expressed in a tissue-specific manner (Supplementary Figure 1A); (3) mutations in this gene are associated with different types of cancer (Supplementary Figure 1B); (4) it appears to be fairly conserved (Supplementary Figure 1C); and (5) with 3 exons it is likely to be processed (Figure 3A). To examine the cell-specific expression pattern of DR5-AS lncRNA, we performed qPCR analyses with total RNAs isolated from two more cell lines, namely MCF7 and Jurkat cells. The expression of DR5-AS in HeLa and Jurkat cells were comparable but much less abundant in MCF7 cells (Figure 3B). Interestingly, cisplatin treatment induced the expression of DR5-AS lncRNA in HeLa and Jurkat cells but not in MCF7 cells (Figure 3C).

The DR5-AS gene is annotated to encode a transcript with 3 exons (Figure 3A). To map the 5′ end 3′ borders of the DR5-AS transcript, we carried out 5′ and 3′ RACE. Our analysis showed that the DR5-AS gene encodes a 2636-nt transcript without a tail (Supplementary Figure 2). Since there appears to be a correlation between the subcellular location of an lncRNA and its regulatory function (Carlevaro-Fita and Johnson, 2019), we examined the intracellular distribution of this transcript. To this extent, qPCR analyses were performed with total, cytoplasmic and nuclear RNAs isolated from cisplatin-treated HeLa cells. To check for the integrity of subcellular RNA fractionation, we used MALAT1 and GAPDH as controls for nuclear and cytoplasmic RNA preparations, respectively (Debacq et al., 2002). In reference to these markers, DR5-AS appears to be localized primarily in the nuclear fraction (Figure 3D).

### DR5-AS lncRNA Modulates Cell Morphology

**FIGURE 4:**
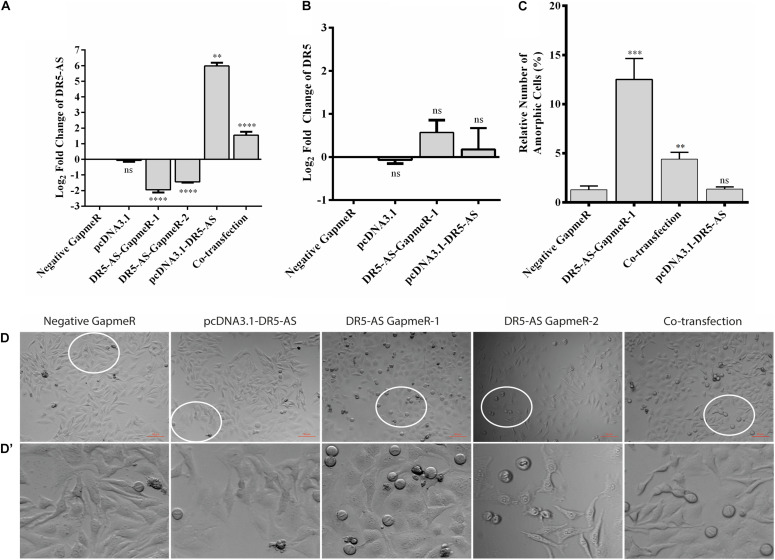
DR5-AS knockdown modulates cell morphology in HeLa cells. **(A)** qPCR analyses of DR5-AS expression. HeLa cells were transfected with various GapmeRs or overexpression vectors for 72 and 48 h respectively and the amount of DR5-AS transcript was measured with qPCR. **(B)** qPCR analyses of DR5 sense mRNA expression. HeLa cells were transfected as explained in Figure 4A and DR5 expression was measured by qPCR. **(C)** Quantitative number of metaphase block cells in DR5-AS knockdown (DR5-AS-GapmeR-1), overexpression (pcDNA3.1-DR5-AS), and co-transfection groups. The same number of cells were visually counted under the microscope from at least three different regions and the percentage of amorphic cells was plotted. **(D)** Brightfield images of transfected cells after incubation period. **(D’)** The magnified areas of the parts encircled in white. Scale bar 50 μm. Negative GapmeR was used as negative control for transfection. pcDNA3.1 represents the empty vector for overexpression. ns, non-significant, *p* > 0.05, ^∗∗^*p* ≤ 0.01, ^∗∗∗^*p* ≤ 0.001, and ^****^p ≤ 0.0001.

The DR5-AS lncRNA gene is physically overlapping with the protein-coding DR5 gene (Figure 3A). The DR5 receptor is known to trigger signal transduction pathways that modulate apoptosis, miRNA biogenesis, survival, and proliferation (Mert and Sanlioglu, 2016). Considering the cisplatin inducibility of DR5-AS lncRNA and its genomic location antisense to the DR5 gene, we hypothesized that the DR5-AS lncRNA could be involved in modulating cell fate. To gain insight into the cellular function of DR5-AS, we exploited a reverse genetics approach to perturb intracellular DR5-AS lncRNA concentration. For this purpose, we employed the GapmeR technology as it has been reported to knock down nuclear lncRNAs more efficiently (Xing et al., 2014). We first quantified the intracellular amount of the DR5-AS lncRNA in HeLa cells 72 h post-transfection with two different Gapmers (DR5-AS-GapmeR-1 and DR5-AS-GapmeR-2). qPCR analyses showed that GapmeR-1 was more efficient in knocking down the DR5-AS lncRNA (Figure 4A). We transfected cells with fluorescent-labeled DR5-AS-GapmeR-1 to examine the transfection efficiency. Fluorescent microscopy showed that more than 60% of HeLa cells were transfected under our transfection conditions (Data not shown). We also cloned the full-length cDNA of DR5-AS lncRNA into pcDNA3.1 to obtain pcDNA3.1-DR5-AS, which was efficiently overexpressed when transfected into HeLa cells (Figure 4A).

A portion of antisense lncRNAs have been reported to regulate transcription of the protein coding gene with which it is overlapping (Magistri et al., 2012). Based on the ENSEMBL entry, there is an 803-bp overlap between the DR5-AS lncRNA and the DR5 protein coding gene. To test the hypothesis that the DR5-AS transcript might transcriptionally regulate DR5 transcription *in cis*, we measured the amount of DR5 mRNA following DR5-AS knockdown and overexpression. Our qPCR analyses showed that DR5-AS knockdown or overexpression was not sufficient to perturb the intracellular DR5 mRNA amount under our experimental conditions (Figure 4B). Although we cannot conclusively eliminate the possibility that the native promoter-driven active transcription of DR5-AS may be required for regulation in *cis*, the mere increase in the DR5-AS transcript abundance does not appear to modulate the intracellular DR5 mRNA concentration. Considering the fact that DR5 overexpression increases the rate of apoptosis in HeLa cells (Poondla et al., 2019), a potential regulation of DR5 by DR5-AS would be expected to modulate the apoptotic rate in HeLa cells.

We then monitored the cell morphology 72 h post-transfection and observed a dramatic change in cell shape upon transfection of HeLa cells with 40 nM of two different versions of unlabeled GapmeR against DR5-AS (Figures 4D,D’). Morphology of the cells transfected with negative GapmeR and pcDNA3.1-DR5-AS remained similar to characteristic adherent HeLa cells exhibiting projections from cytoplasm, while cells transfected with DR5-AS GapmeR-1, but not quite with DR5-AS GapmeR-2 were transformed into spherical-shaped adherent cells. These cells appeared to have been arrested at the metaphase as revealed by DAPI staining (Supplementary Figure 3). Approximately 12% of the cells possess this phenotype (Figure 4C). To ensure that the change in cell morphology is specifically due to the knockdown of DR5-AS, we also performed co-transfection assays with the DR5-AS overexpression construct, pcDNA3.1-DR5-AS. Although the overexpression of HeLa cells with pcDNA3.1-DR5-AS did not yield any observable cellular phenotype (Figure 4D), DR5-AS lncRNA overexpression was able to partially rescue the GapmeR-induced morphological change (Figures 4D,D’).

### DR5-AS Knockdown Perturbs the Transcriptome Associated With Cell Proliferation and Cell Cycle

Cisplatin is known to exert a pleiotropic effect on cells (Dasari and Bernard Tchounwou, 2014). Based on the physical overlap between DR5-AS and DR5 with respect to their genomic location, it is plausible to suggest that DR5-AS may function through stress, proliferation, apoptosis, drug metabolism or cell motility, all of which are known to be modulated by cisplatin treatment.

**FIGURE 5:**
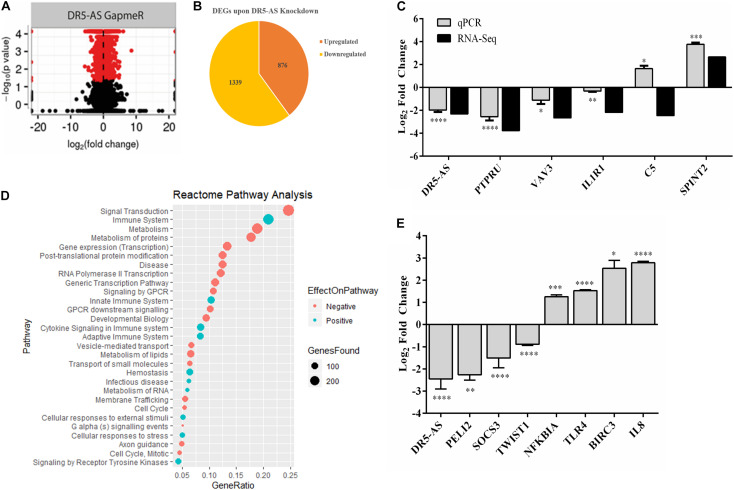
DR5-AS-knockdown-mediated perturbations in gene expression. Total RNAs isolated from HeLa cells transfected with negative GapmeR or DR5-AS GapmeR were subjected to RNA-seq. A volcano plot and pie chart of differentially expressed genes after knockdown are presented in **(A,B)**, respectively. **(C)** qPCR validation of some genes differentially expressed in DR5-AS knockdown HeLa cells. **(D)** Reactome Pathway analysis of genes differentially expressed in HeLa cells transfected with DR5-AS GapmeR. **(E)** qPCR analyses of immune system-related genes as determined to be differentially expressed based on the Reactome pathway analysis. ^∗^*p* ≤ 0.05, ^∗∗^*p* ≤ 0.01, ^∗∗∗^*p* ≤ 0.001, and ^****^p ≤ 0.0001.

To gain insight into the cellular function of the DR5-AS transcript, we exploited the power of the transcriptomics approach. To this end, we first knocked down the DR5-AS transcript with GapmeR-1 and sequenced the total RNAs isolated from these cells in parallel to those isolated from control-GapmeR-transfected HeLa cells. Bioinformatics analyses revealed the differential expression of 2,215 mRNAs, of which 876 and 1,339 up- and down-regulated, respectively (Figures 5A,B). We then performed a Reactome pathway analysis to deduce biological processes affected by DR5-AS knockdown (Figure 5D). We also performed a Gene Ontology analysis to examine biological processes affected upon DR5-AS knockdown. Interestingly, the most affected biological processes included, but are not limited to, basement membrane organization, cell migration, collagen fibril organization, coagulation, cell shape, and proliferation (Supplementary Table 1). Many of these cellular biological processes could potentially be the cause of the change in cell morphology presented in Figure 4. Thus, we validated by qPCR the amount of some DEGs associated with cell proliferation and cell cycle. The qPCR results were in congruous with the RNA-seq data except for C5 (Figure 5C). Strikingly, the Reactome pathway and Gene Ontology analyses showed that DR5-AS is very likely to be involved in immune system-related cellular processes (Figure 5D). Our qPCR analyses validated the differential expression of immune system-related genes such as SOCS3, TLR4, and IL8 in DR5-AS knockdown HeLa cells (Figure 5E).

### DR5-AS Knockdown Reduces Cell Proliferation and Metastasis in HeLa Cells

**FIGURE 6:**
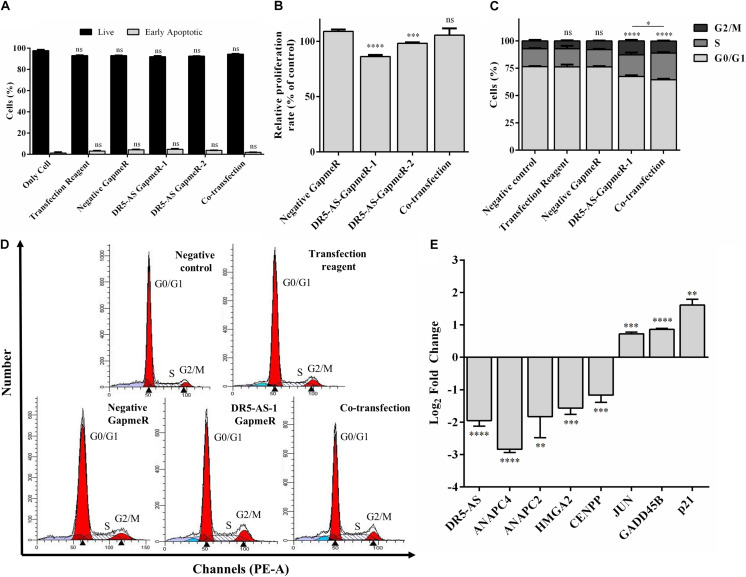
DR5-AS knockdown modulates proliferation and cell cycle in HeLa cells. **(A)** The rate of early apoptosis in HeLa cells under various transfection conditions was determined by flow cytometry. HeLa cells were transfected with DR5-AS GapmeR alone (DR5-AS GapmeR-1 and DR5-AS GapmeR-2) or in combination of DR5-AS GapmeR-1 with pcDNA3.1-DR5-AS (co-transfection). Only cell (no transfection), transfection reagent (no GapmeR or plasmid) and negative GapmeR were used as controls. 72 h post-transfection, the rate of early apoptotic cells was determined by Annexin V/7AAD staining. The stained cells were analyzed by a flow cytometer. **(B)** Proliferation rate of HeLa cells transfected with DR5-AS GapmeR alone (DR5-AS GapmeR-1 and DR5-AS GapmeR-2) or in combination of DR5-AS GapmeR-1 with pcDNA3.1-DR5-AS (cotransfection) for 72 h. Untransfected (Negative control) and transfected (Transfection Reagent, Negative GapmeR, DR5-AS-GapmeR-1 or co-transfection) HeLa cells described in Figure 4C were also subjected to cell cycle analysis and the percent of cells in each phase **(C)** was calculated from DNA histograms **(D)**. **(E)** qPCR analyses of genes associated with cell cycle in DR5-AS knockdown HeLa cells (Negative GapmeR versus DR5-AS-GapmeR-1). ns, non-significant (*p* > 0.05), *p ≤ 0.05, **p ≤ 0.01, ***p ≤ 0.001, ****p ≤ 0.0001.

To functionally test the observations made by the transcriptomics approach, we first checked the viability of cells with morphological changes to eliminate the possibility that these cells are going through cell death. Our flow cytometric analyses showed that the percentage of Annexin V-positive cells were quite comparable among GapmeR-, pcDNA3.1.DR5-AS- and co-transfected cells, indicating that these cells are indeed viable (Figure 6A). We also measured the intracellular uptake of NucRed^TM^ Dead 647 ReadyProbes^TM^ (Thermo Fisher Scientific), a fluorescent dye used as a marker for dead cells. The NucRed^TM^ Dead 647 ReadyProbes^TM^ fluorescent dye penetrated into neither negative control GapmeR- nor DR5-AS-GapmeR-transfected HeLa cells, further confirming the viability of these cells (Supplementary Figure 4). We then measured the proliferation rate of HeLa cells following 72 h transfection with negative and DR5-AS GapmeR. In agreement with the transcriptomics data, we observed a 22.7% reduction in the proliferation rate of HeLa cells upon DR5-AS knockdown in comparison with the cells transfected with negative GapmeR (Figure 6B). There was a correlation between the knockdown efficiency of GapmeR-1 and 2 (Figure 4A) and the corresponding reduced proliferation rate. To ensure that the GapmeR-mediated knockdown was responsible for this reduction in proliferation rate, we tried to rescue the phenotype by overexpressing DR5-AS lncRNA. As expected, the overexpression of DR5-AS has partially rescued the DR5-AS-GapmeR-mediated decrease in the proliferation rate (Figure 6B).

**FIGURE 7:**
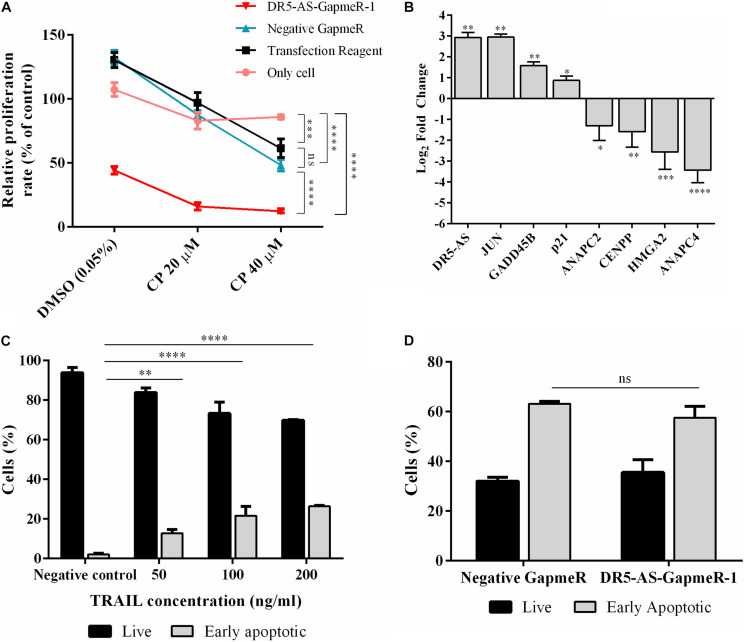
Cisplatin- and TRAIL-induced cellular changes in DR5-AS knockdown HeLa cells. **(A)** Proliferation rate of cisplatin-treated HeLa cells transfected with DR5-AS GapmeR. HeLa cells were transfected with DR5-AS GapmeR-1 for 72 h and then treated with cisplatin for additional 16 h. Proliferation rate of cells was measured by WST-1 assay. “Only cell” (no transfection), “transfection reagent” (cells transfected with transfection reagent only), and negative GapmeR were used as controls. **(B)** qPCR analyses of cell cycle-associated genes in cisplatin-treated HeLa cells. HeLa cells were treated with 80 μM cisplatin for 16 h and qPCR analyses were carried out with total RNAs isolated from control (0.1% DMSO) and cisplatin-treated cells. **(C)** Dose response of HeLa cells treated with TRAIL for 3 h treatment. **(D)** Apoptosis rate of HeLa cells after TRAIL treatment. HeLa cells were treated with 200 ng/ml TRAIL for 3 h. The rate of apoptosis was measured by flow cytometry following Annexin V/7AAD staining. ns, non-significant, *p* > 0.05, ^∗^*p* ≤ 0.05, ^∗∗^*p* ≤ 0.01, ^∗∗∗^*p* ≤ 0.001, and ^****^p ≤ 0.0001.

We extended our functional analysis to cover cell cycle analyses as well since DR5-AS knockdown perturbed the gene expression pattern associated with cell cycle (Figure 5D). Thus, we performed a post-knockdown flow cytometric cell cycle analysis. Although the transfection reagent or the negative GapmeR did not cause any discernible difference in the cell cycle profile, DR5-AS knockdown caused the cells to shift from the G0/G1 phase to the S and G2/M phases (Figures 6C,D). Although DR5-AS overexpression did not completely rescue the DR5-AS-knockdown-mediated shift, it has reduced the percentage of the cells in the G2/M phase from 12.7% to 11%. We then selected a number of DEGs associated with cell cycle that were identified through the reactome pathway analyses (Figure 5D) for validation by qPCR. Parallel to RNA-seq data, DR5-AS knockdown induced p21 and GADD45B and down-regulated the expression of ANAPC2 and ANAPC4 which are subunits of anaphase promoting complex/cyclosome (APC/C) and CENPP, as key regulators of cell cycle (Figure 6E; Stein and Pardee, 2004). To test whether DR5-AS knockdown exacerbates the antiproliferative effect of cisplatin, we first transfected HeLa cells with the DR5-AS and then treated with relatively milder concentrations of cisplatin (e.g., 20 and 40 μM). DR5-AS knockdown exacerbated the antiproliferative effect of cisplatin nearly 3.2-fold (Figure 7A). This observation has prompted us to check whether or not cisplatin- and DR5-AS-mediated reduction in proliferation rate involve the differential expression of similar genes. Strikingly, cisplatin treatment in HeLa cells perturbed the expression of similar cell cycle-related genes albeit to a different extent (Figure 7B). Although DR5-AS knockdown does not alter the rate of apoptosis in HeLa cells (Figure 6A), we postulated that DR5-AS could mitigate the TRAIL-induced apoptosis due to their overlapping genomic context (Figure 3A). Thus, we examined the effect of DR5-AS knockdown on TRAIL-induced apoptosis. Under our experimental condition, DR5-AS knockdown had no effect on TRAIL-induced apoptosis (Figures 7C,D).

**FIGURE 8:**
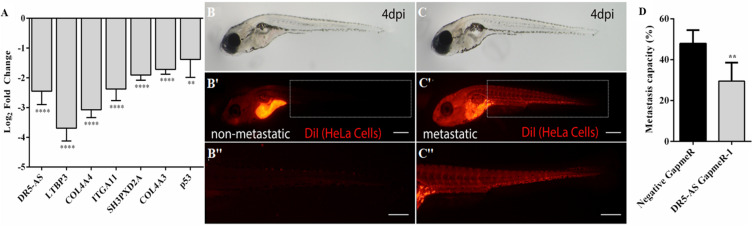
Analysis of metastasis rate in zebrafish xenograft assay. **(A)** qPCR analysis of metastasis-related genes in HeLa cells transfected with DR5-AS GapmeR-1. DR5-AS was knocked down in HeLa cells by transfecting the cells with DR5-AS GapmeR-1 for 72 h. qPCR was carried out with total RNAs isolated from cells transfected with negative GapmeR and DR5-AS GapmeR-1. To examine the metastasis rate in the zebrafish xenograft assay **(B–C”)**, control cells and DR5-AS GapmeR treated cells were injected to the yolk of 2 dpf zebrafish. **(B–B”)** Xenografts with non-metastatic tumors display local tumors at the injection site **(B’)**. No tumor cells are detectable in the body **(B”)**. In metastatic xenografts **(C–C”)**, tumor cells are detected in the injection site **(C’)** and rest of the body (**C”** a closeup view of cells in the tail region). **(D)** Percentage of metastatic larvae is 47.9% in control group whereas it is 29.5% in DR5-AS GapmeR group. The graph is plotted based on average of 3 replicates of each cell type (*n* = 18–20 per set), and 2 independent experiments. Dashed boxes in (**B’**,**C’)** indicate the regions imaged in **(B”,C”)**, respectively. Scale bars represent 500 μm. Images were recorded with Olympus SZX16 stereomicroscope. **p ≤ 0.01, ****p ≤ 0.0001.

In order to examine the functional effect of DR5-AS knockdown *in vivo*, we exploited the well-established zebrafish larval xenograft model (Brown et al., 2017; Hason and Bartůněk, 2019). This model allows *in vivo* quantitation of invasion and metastasis of human cancer cells in an intact live organism. We first carried out a transwell assay to check the effect of DR5-AS knockdown on migration at the cellular level. Despite the differential expression of genes associated with cell migration and metastasis based on the Reactome and Gene Ontology analyses (Figure 5D and Supplementary Table 1), we detected no change in the rate of migration upon DR5-AS knockdown in HeLa cells (Data not shown). However, the expression of genes associated with extracellular matrix and metastatis were altered upon DR5-AS knockdown as determined by qPCR analyses (Figure 8A). Thus, we decided to examine the effect of DR5-AS knockdown *in vivo*. To this extent, we transfected HeLa cells with negative and DR5-AS GapmeR for 24 h to generate control and DR5-AS knockdown HeLa cells, which were live stained with a red fluorescent membrane dye for *in vivo* imaging (Iscan et al., 2021). We induced tumor formation locally in the yolk sac of 2 days post fertilization (dpf) wild type zebrafish, by xenotransplantation. The xenografted zebrafish larvae were monitored for the next 4 days and the metastasis rate was quantified at 4 dpi. In both groups we observed larvae with local tumors (i.e., all cells remained in the original injection site) (Figures 8B–B”), or metastatic tumors in which the cells invaded the blood vessels and spread to the tail/trunk/head regions of the host larvae (Figures 8C–C”). After quantifying the number of metastatic and local tumors in both groups, we showed that the silencing of DR5-AS caused a significant reduction of metastasis rate of HeLa cells from 47.9% to 29.5% (Figure 8D).

## Discussion

We provide the first comprehensive expression profile of lncRNAs in cisplatin-treated HeLa cells. Under our experimental setting, we identified 10,214 de-regulated lncRNAs that include not only the well-known examples of lincRNAs and antisense ncRNAs but also an interesting repertoire of 155 intronic lncRNAs. We selected DR5-AS for further functional analysis because of its genomic location being antisense to the death receptor (DR) 5, a TRAIL-bound receptor that modulates cell death and survival (Kimberley and Screaton, 2004). GapmeR-mediated silencing of the nuclear DR5-AS transcript caused a change in cell morphology without affecting cell viability, which could be partially rescued upon its ectopic overexpression. Transcriptomics analyses of the DR5-AS knockdown HeLa cells uncovered changes in the expression of genes associated with cell morphology, cell proliferation, cell cycle, and migration. Congruently, DR5-AS knockdown led to a drop in the proliferation rate and brought about a shift in the cell cycle as evidenced by an arrest at S and G2/M phases. Zebrafish xenograft experiment showed the reduced metastatic capacity of DR5-AS knockdown HeLa cells.

Previous microarray studies have shown that cisplatin modulates the expression of lncRNAs in various cisplatin-treated cell lines such as A549 lung adenocarcinoma cell line (Yang et al., 2013; Hu et al., 2017) and CAL-27 and SSC-9 tongue squamous carcinoma cell lines (Fan et al., 2020). In addition, bioinformatics methods were employed to examine lncRNAs associated with platinum drugs in high grade serous ovarian cancer (Liu et al., 2017). Interestingly, a pan-cancer analysis of RNA-seq data of 648 samples from 11 different cancer types revealed cancer-specific lncRNA expression upon cisplatin treatment. Subsequent functional analyses have shown that lncRNAs could function through various mechanisms to modulate cisplatin-modulated cellular responses. For example, cisplatin-sensitivity-associated lncRNA (CISAL) inhibits BRCA1 transcription and thereby controls cisplatin sensitivity in squamous cell carcinoma (Fan et al., 2020). LncRNA AK126698 was reported to be involved in Wnt/B-catenin-mediated regulation of cisplatin-induced apoptosis in A549 cells (Yang et al., 2013). LncRNA TUG1, on the other hand, promotes cisplatin resistance through the epigenetic regulation of miR-194-5p in bladder cancer (Yu et al., 2019). Although microarray-based transcriptomics approaches have been highly fruitful in identification of especially highly expressed lncRNAs, they are limited to the analysis of known lncRNAs. We employed an RNA sequencing approach to ensure a more comprehensive coverage. Indeed, our analyses revealed the stable accumulation of a number of intron-derived lncRNAs.

Antisense lncRNAs have been reported to regulate the expression of nearby genes in *cis* or *trans* (Magistri et al., 2012). In addition to the regulation of transcription-related processes, NATs can modulate gene expression through RNA:DNA or RNA:RNA interactions in the nucleus or RNA:RNA interactions in the cytosol (Faghihi and Wahlestedt, 2009). We prioritized DR5-AS for functional analyses as it is positioned antisense to the DR5 receptor. TRAIL, which is an anticancer agent in human, triggers apoptosis by ligating to the DR5 receptor (Yagita et al., 2004), making DR5-AS a prominent choice for studying cisplatin’s downstream effects. Although DR5-AS is located in the nucleus (Figure 3), neither its ectopic overexpression nor its GapmeR-mediated knockdown modulates DR5 expression under our experimental setting (Figure 4B). Additionally, DR5-AS knockdown had no effect on TRAIL-induced apoptotis (Figure 7D). These observations suggest that either DR5-AS regulates its target genes in *trans* or its in-cis transcription is required to regulate the sense mRNA.

We exploited reverse genetics to gain insight into the potential function of DR5-AS. To this extent, we employed GapmeR technology as GapmeRs are more efficient in knocking down nuclear lncRNAs compared to siRNAs (Xing et al., 2014). Interestingly, knocking down DR5-AS caused a severe change in cell morphology in HeLa cells (Figures 4D,D’), which could be partially rescued by overexpression of DR5-AS. These round-shaped cells maintained adherence to the culture flasks and were alive as evident by flow cytometric analysis of cells (Figure 6A) and by microscopic observation after NucRed^TM^ Dead 647 ReadyProbes^TM^ staining (Supplementary Figure 4). The decrease in metastatic behavior of HeLa cells upon DR5-AS silencing may be in part due to the change in cell morphology. While the round shaped cells may loose contact with neighboring cells potentially favoring dissemination, motile tumor cells often display a mesenchymal elongated morphology as motility requires formation of active protrusions at the leading edge of the cell to ensure migration (Gulvady et al., 2018; Baskaran et al., 2020). Gene Ontology analysis showed perturbation of gene expression patterns associated with basement membrane organization and cell migration along with collagen fibril organization (Supplementary Table 1). It requires further investigation whether the potential change in basement membrane organization is associated with the reduced metastatic behavior. Surprisingly, RNA-seq analysis of total RNAs from DR5-AS knockdown cells revealed modulation of immune-system-related genes (Figure 5D) in HeLa cells of epithelial morphology. Considering the GapmeR-mediated morphological change and relevance to cisplatin’s effects, we noticed the changes in gene expression associated with cell cycle and proliferation as well. Expectedly, DR5-AS knockdown resulted in a decrease in the proliferation rate of HeLa cells coupled with a cell cycle arrest (Figure 6). It is highly interesting that DR5-AS knockdown reduces proliferation rate and causes a cell cycle arrest without triggering cell death. This phenomenon is typically seen in the immune system. For example, increased cell proliferation, without a change in cell death, has been reported to induce lymphocytosis in bovine leukemia virus-infected sheep (Debacq et al., 2002). Taking into account the extent of affected immune system-related genes following DR5-AS knockdown (Figure 5E), it would be interesting to probe into the potential function of DR5-AS in regulating innate or adaptive immunity.

## Data Availability Statement

The datasets presented in this study can be found in online repositories. The names of the repository/repositories and accession number(s) can be found below: https://www.ncbi.nlm.nih.gov/geo/, GSE160227 and https://www.ncbi.nlm.nih.gov/geo/, GSE165560.

## Ethics Statement

The animal study was reviewed and approved by Izmir Biomedicine and Genome Center Local Animal Studies Ethics Committee.

## Author Contributions

BA contemplated the project. GC-A designed. MB and GC-A performed the zebrafish xenograft experiment. DCG, İE, UA, and OS performed all other experiments. DCG performed RNA-seq data analyses. DCG, İE, and BA wrote the manuscript. All authors contributed to the article and approved the submitted version.

## Conflict of Interest

The authors declare that the research was conducted in the absence of any commercial or financial relationships that could be construed as a potential conflict of interest.

## Publisher’s Note

All claims expressed in this article are solely those of the authors and do not necessarily represent those of their affiliated organizations, or those of the publisher, the editors and the reviewers. Any product that may be evaluated in this article, or claim that may be made by its manufacturer, is not guaranteed or endorsed by the publisher.

## Footnotes

1
https://www.fasteris.com/dna/
