# Supplementary material for: Transcriptomics Profiling Identifies Cisplatin-Inducible Death Receptor 5 Antisense Long Non-coding RNA as a Modulator of Proliferation and Metastasis in HeLa Cells
Source: Front Cell Dev Biol. 2021 Aug 23;9:688855. doi: 10.3389/fcell.2021.688855 (PMC8419520; doi:10.3389/fcell.2021.688855)
Supplement: Supplementary file 1 [file Data_Sheet_1.docx]

**Gürer et al. Supplementary Figure 1**


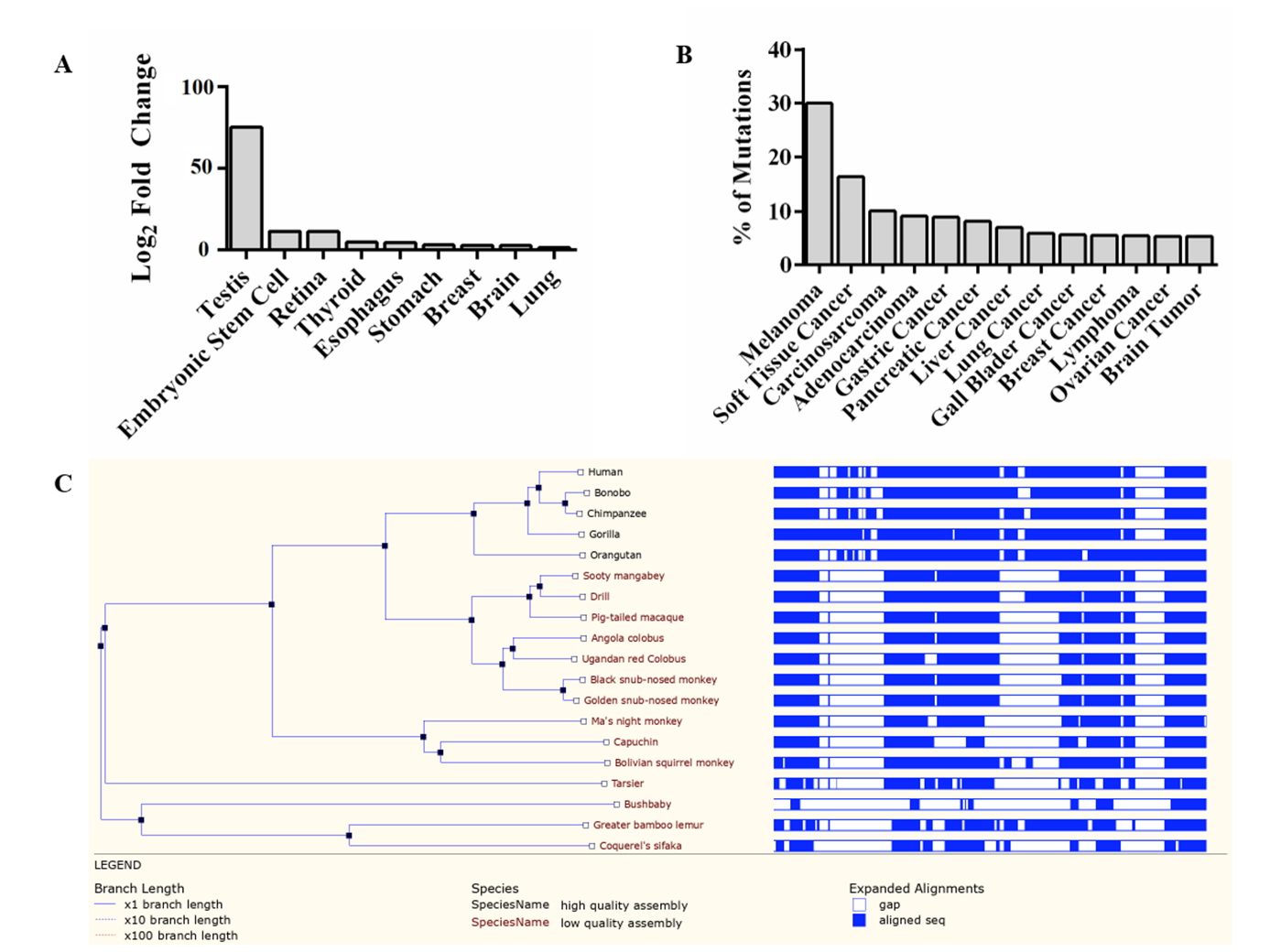


Supplementary Figure 1. A. Differential expression and B. Percentage of mutations observed in DR5-AS in different cancer cases. C. MSA analysis of DR5-AS by 27 primates EPO-Low-Coverage.

**Gürer et al. Supplementary Figure 2**


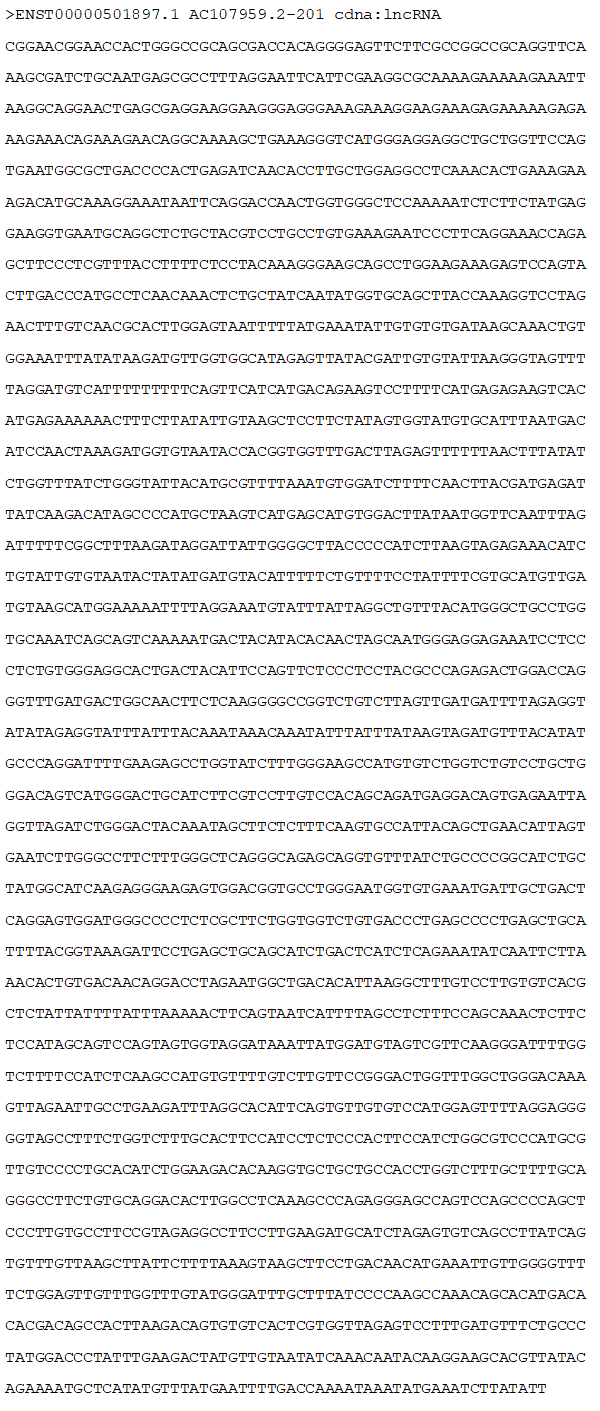


Supplementary Figure 2. Full-length cDNA sequence of DR5-AS.

**Gürer et al. Supplementary Figure 3**


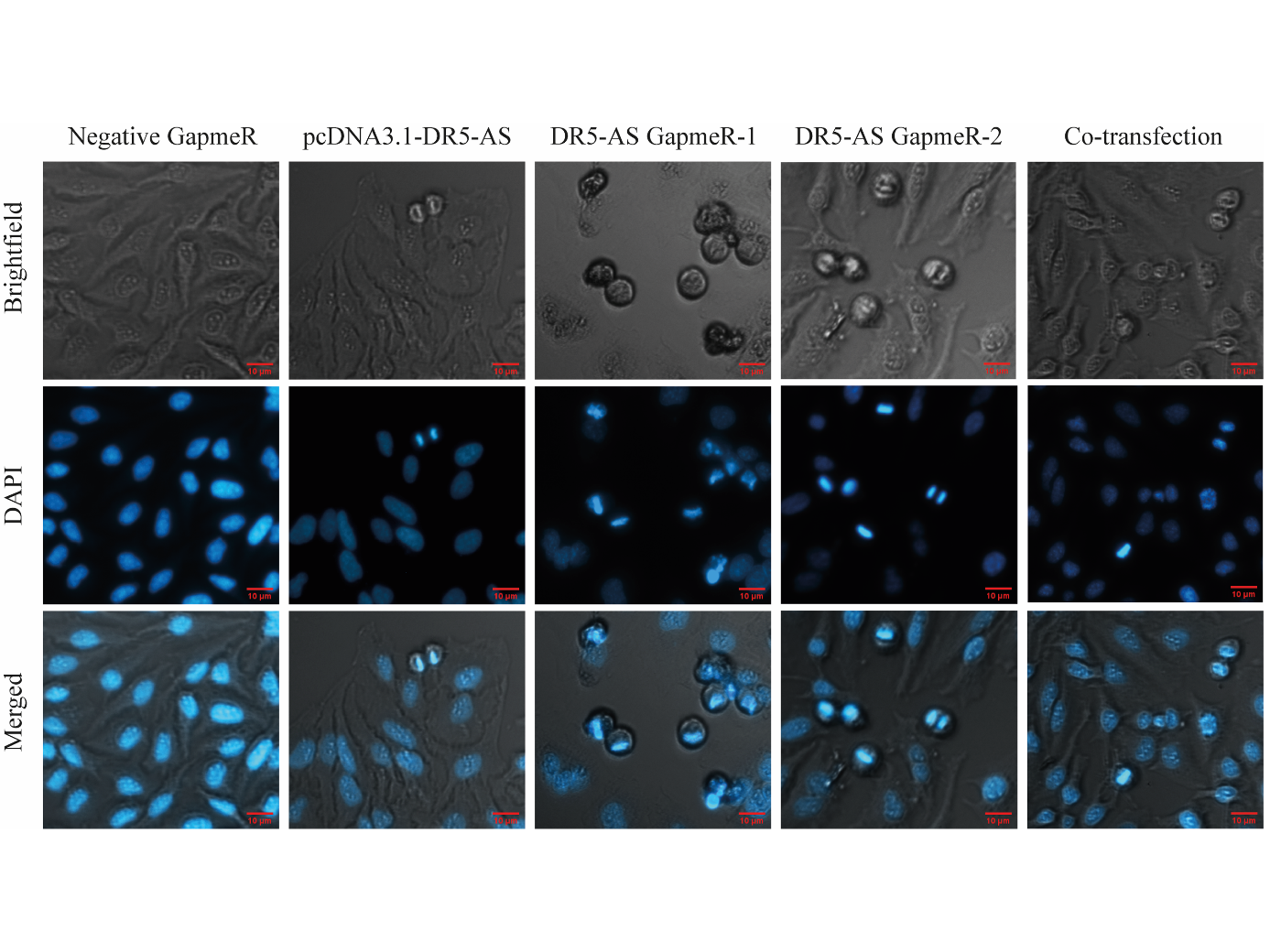


Supplementary Figure 3. Phenotypic observation of DR5-AS-silenced HeLa cells stained with DAPI. Negative Gapmer was used as a negative control. Magnification is 10X

**Gürer et al. Supplementary Figure 4**


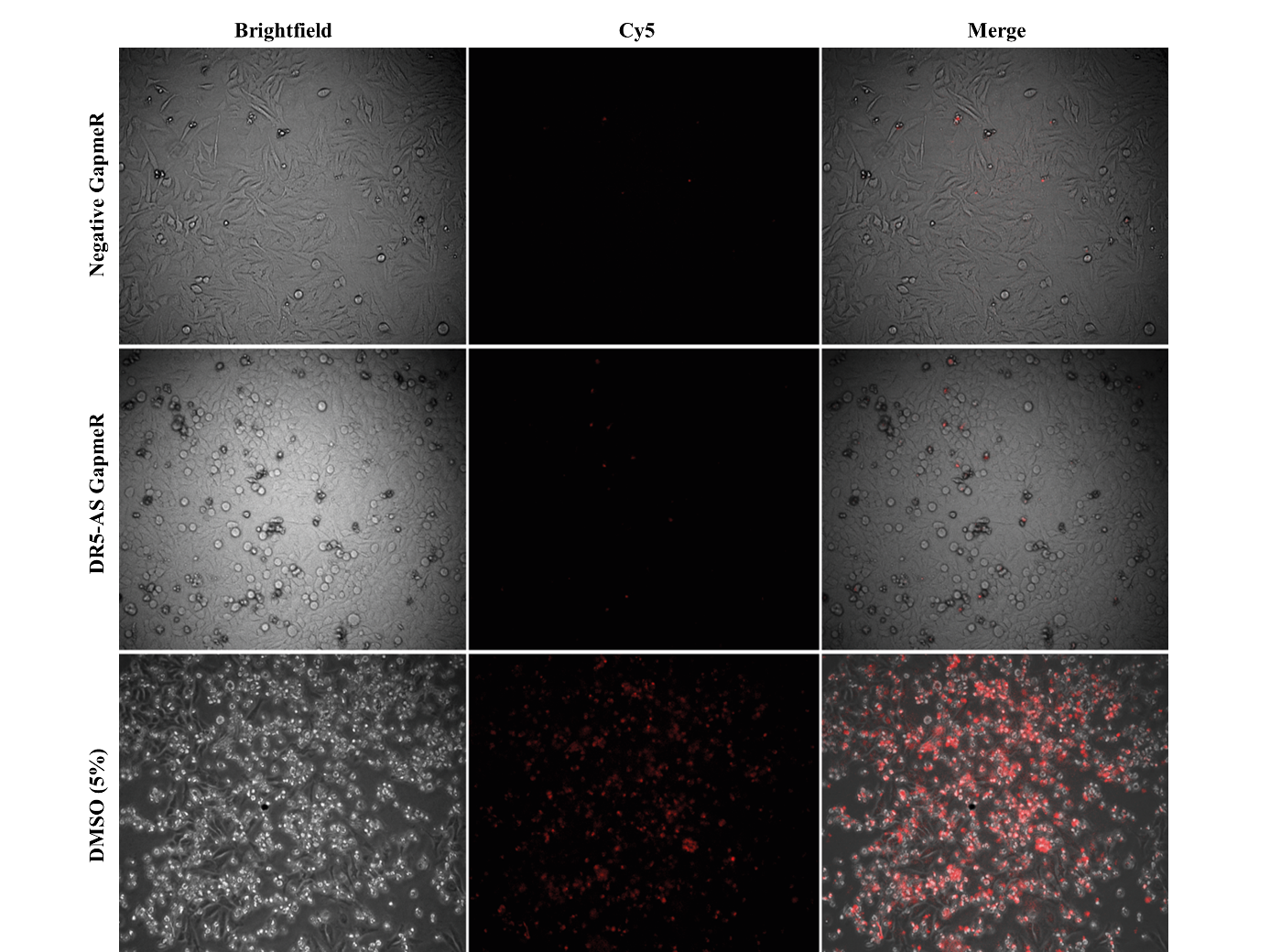


Supplementary Figure 4. Phenotypic microscopic analysis of HeLa cells transfected with DR5-AS GapmeR-1. Cells were transfected as explained in Materials and Methods. Live cell images were taken under a fluorescent microscope after staining cells with NucRed™ Dead 647 ReadyProbes™. DMSO (5%) was used as positive control. Magnification is 10X.

**Gürer et al. Supplementary Table 1.** Gene Ontology Analysis of DR-AS-silenced HeLa cells

| **Analysis Type:** | **PANTHER Overrepresentation Test (Released 20210224)** | | | | | | |
| --- | --- | --- | --- | --- | --- | --- | --- |
| **Annotation Version and Release Date:** | **GO Ontology database DOI: 10.5281/zenodo.4495804 Released 2021-02-01** | | | | | | |
| **Analyzed List:** | **upload_1 (Homo sapiens)** | | | | | | |
| **Reference List:** | **Homo sapiens (all genes in database)** | | | | | | |
| **Test Type:** | **FISHER** | | | | | | |
| **Correction:** | **FDR** | | | | | | |
| **GO biological process complete** | **Human - REFLIST (20595)** | **DR5-AS (1864)** | **expected** | **over/under** | **fold Enrichment** | **raw P-value** | **FDR** |
| basement membrane organization (GO:0071711) | 27 | 10 | 2,44 | + | 4,09 | 6,55E-04 | 4,99E-02 |
| negative regulation of epithelial cell apoptotic process (GO:1904036) | 46 | 14 | 4,16 | + | 3,36 | 3,18E-04 | 2,80E-02 |
| regulation of smooth muscle cell migration (GO:0014910) | 61 | 18 | 5,52 | + | 3,26 | 6,55E-05 | 8,40E-03 |
| regulation of pri-miRNA transcription by RNA polymerase II (GO:1902893) | 53 | 15 | 4,8 | + | 3,13 | 3,78E-04 | 3,07E-02 |
| negative regulation of coagulation (GO:0050819) | 56 | 15 | 5,07 | + | 2,96 | 6,15E-04 | 4,74E-02 |
| regulation of blood coagulation (GO:0030193) | 71 | 18 | 6,43 | + | 2,8 | 3,25E-04 | 2,80E-02 |
| collagen fibril organization (GO:0030199) | 103 | 26 | 9,32 | + | 2,79 | 1,84E-05 | 2,77E-03 |
| regulation of coagulation (GO:0050818) | 76 | 19 | 6,88 | + | 2,76 | 2,62E-04 | 2,41E-02 |
| positive regulation of lipid localization (GO:1905954) | 112 | 26 | 10,14 | + | 2,56 | 6,81E-05 | 8,66E-03 |
| unsaturated fatty acid metabolic process (GO:0033559) | 103 | 23 | 9,32 | + | 2,47 | 2,78E-04 | 2,54E-02 |
| digestive tract development (GO:0048565) | 125 | 27 | 11,31 | + | 2,39 | 1,55E-04 | 1,58E-02 |
| regulation of phosphatidylinositol 3-kinase signaling (GO:0014066) | 126 | 27 | 11,4 | + | 2,37 | 1,69E-04 | 1,68E-02 |
| regulation of wound healing (GO:0061041) | 131 | 28 | 11,86 | + | 2,36 | 1,29E-04 | 1,37E-02 |
| response to hydrogen peroxide (GO:0042542) | 119 | 25 | 10,77 | + | 2,32 | 3,74E-04 | 3,07E-02 |
| fatty acid biosynthetic process (GO:0006633) | 124 | 26 | 11,22 | + | 2,32 | 2,86E-04 | 2,59E-02 |
| regulation of response to wounding (GO:1903034) | 162 | 33 | 14,66 | + | 2,25 | 7,36E-05 | 8,99E-03 |
| digestive system development (GO:0055123) | 134 | 27 | 12,13 | + | 2,23 | 4,41E-04 | 3,48E-02 |
| blood vessel development (GO:0001568) | 489 | 90 | 44,26 | + | 2,03 | 6,44E-09 | 3,08E-06 |
| vasculature development (GO:0001944) | 512 | 92 | 46,34 | + | 1,99 | 1,33E-08 | 5,11E-06 |
| blood vessel morphogenesis (GO:0048514) | 406 | 70 | 36,75 | + | 1,9 | 2,97E-06 | 6,79E-04 |
| response to drug (GO:0042493) | 383 | 65 | 34,66 | + | 1,88 | 8,71E-06 | 1,51E-03 |
| angiogenesis (GO:0001525) | 314 | 53 | 28,42 | + | 1,86 | 7,71E-05 | 9,28E-03 |
| fatty acid metabolic process (GO:0006631) | 327 | 55 | 29,6 | + | 1,86 | 7,13E-05 | 8,79E-03 |
| negative regulation of protein phosphorylation (GO:0001933) | 351 | 58 | 31,77 | + | 1,83 | 5,87E-05 | 7,59E-03 |
| tube morphogenesis (GO:0035239) | 645 | 106 | 58,38 | + | 1,82 | 5,66E-08 | 1,86E-05 |
| renal system development (GO:0072001) | 288 | 47 | 26,07 | + | 1,8 | 4,19E-04 | 3,38E-02 |
| negative regulation of phosphorylation (GO:0042326) | 395 | 64 | 35,75 | + | 1,79 | 3,67E-05 | 4,99E-03 |
| tube development (GO:0035295) | 841 | 136 | 76,12 | + | 1,79 | 1,58E-09 | 9,57E-07 |
| positive regulation of cell migration (GO:0030335) | 518 | 83 | 46,88 | + | 1,77 | 4,08E-06 | 8,58E-04 |
| positive regulation of locomotion (GO:0040017) | 558 | 89 | 50,5 | + | 1,76 | 2,17E-06 | 5,18E-04 |
| circulatory system development (GO:0072359) | 853 | 134 | 77,2 | + | 1,74 | 1,14E-08 | 4,61E-06 |
| positive regulation of cell motility (GO:2000147) | 543 | 85 | 49,15 | + | 1,73 | 6,70E-06 | 1,20E-03 |
| positive regulation of cellular component movement (GO:0051272) | 556 | 87 | 50,32 | + | 1,73 | 5,86E-06 | 1,14E-03 |
| urogenital system development (GO:0001655) | 320 | 50 | 28,96 | + | 1,73 | 5,82E-04 | 4,52E-02 |
| regulation of cell migration (GO:0030334) | 885 | 138 | 80,1 | + | 1,72 | 1,02E-08 | 4,35E-06 |
| regulation of locomotion (GO:0040012) | 990 | 152 | 89,6 | + | 1,7 | 4,20E-09 | 2,21E-06 |
| regulation of cell motility (GO:2000145) | 947 | 144 | 85,71 | + | 1,68 | 2,05E-08 | 7,69E-06 |
| regulation of lipid metabolic process (GO:0019216) | 403 | 61 | 36,47 | + | 1,67 | 3,29E-04 | 2,82E-02 |
| glycerolipid metabolic process (GO:0046486) | 400 | 60 | 36,2 | + | 1,66 | 4,38E-04 | 3,47E-02 |
| circulatory system process (GO:0003013) | 476 | 71 | 43,08 | + | 1,65 | 1,54E-04 | 1,58E-02 |
| cell projection morphogenesis (GO:0048858) | 503 | 75 | 45,53 | + | 1,65 | 1,21E-04 | 1,31E-02 |
| regulation of cellular component movement (GO:0051270) | 1026 | 152 | 92,86 | + | 1,64 | 3,54E-08 | 1,21E-05 |
| negative regulation of phosphate metabolic process (GO:0045936) | 513 | 76 | 46,43 | + | 1,64 | 1,07E-04 | 1,19E-02 |
| monocarboxylic acid metabolic process (GO:0032787) | 514 | 76 | 46,52 | + | 1,63 | 1,41E-04 | 1,48E-02 |
| negative regulation of phosphorus metabolic process (GO:0010563) | 514 | 76 | 46,52 | + | 1,63 | 1,41E-04 | 1,47E-02 |
| regulation of cell adhesion (GO:0030155) | 726 | 107 | 65,71 | + | 1,63 | 5,01E-06 | 9,87E-04 |
| cellular response to growth factor stimulus (GO:0071363) | 503 | 74 | 45,53 | + | 1,63 | 1,67E-04 | 1,68E-02 |
| negative regulation of cell population proliferation (GO:0008285) | 689 | 101 | 62,36 | + | 1,62 | 1,31E-05 | 2,11E-03 |
| morphogenesis of an epithelium (GO:0002009) | 430 | 63 | 38,92 | + | 1,62 | 6,59E-04 | 5,00E-02 |
| plasma membrane bounded cell projection morphogenesis (GO:0120039) | 499 | 73 | 45,16 | + | 1,62 | 2,15E-04 | 2,06E-02 |
| lipid biosynthetic process (GO:0008610) | 598 | 87 | 54,12 | + | 1,61 | 7,43E-05 | 9,01E-03 |
| neuron projection morphogenesis (GO:0048812) | 495 | 72 | 44,8 | + | 1,61 | 2,75E-04 | 2,52E-02 |
| anatomical structure morphogenesis (GO:0009653) | 2135 | 310 | 193,23 | + | 1,6 | 4,15E-15 | 6,55E-11 |
| supramolecular fiber organization (GO:0097435) | 597 | 86 | 54,03 | + | 1,59 | 9,90E-05 | 1,12E-02 |
| cellular component morphogenesis (GO:0032989) | 604 | 87 | 54,67 | + | 1,59 | 8,31E-05 | 9,86E-03 |
| cell part morphogenesis (GO:0032990) | 522 | 75 | 47,24 | + | 1,59 | 2,90E-04 | 2,60E-02 |
| regulation of protein kinase activity (GO:0045859) | 797 | 114 | 72,13 | + | 1,58 | 9,17E-06 | 1,57E-03 |
| response to growth factor (GO:0070848) | 532 | 76 | 48,15 | + | 1,58 | 3,25E-04 | 2,83E-02 |
| tissue morphogenesis (GO:0048729) | 532 | 76 | 48,15 | + | 1,58 | 3,25E-04 | 2,82E-02 |
| embryonic morphogenesis (GO:0048598) | 561 | 80 | 50,77 | + | 1,58 | 2,61E-04 | 2,42E-02 |
| cellular lipid metabolic process (GO:0044255) | 975 | 139 | 88,24 | + | 1,58 | 1,11E-06 | 2,96E-04 |
| cell morphogenesis (GO:0000902) | 711 | 101 | 64,35 | + | 1,57 | 4,09E-05 | 5,47E-03 |
| cell morphogenesis involved in differentiation (GO:0000904) | 554 | 78 | 50,14 | + | 1,56 | 4,20E-04 | 3,36E-02 |
| positive regulation of developmental process (GO:0051094) | 1259 | 177 | 113,95 | + | 1,55 | 6,26E-08 | 2,01E-05 |
| neuron projection development (GO:0031175) | 683 | 96 | 61,82 | + | 1,55 | 8,96E-05 | 1,05E-02 |
| enzyme linked receptor protein signaling pathway (GO:0007167) | 726 | 102 | 65,71 | + | 1,55 | 4,98E-05 | 6,60E-03 |
| negative regulation of developmental process (GO:0051093) | 870 | 121 | 78,74 | + | 1,54 | 1,58E-05 | 2,44E-03 |
| regulation of kinase activity (GO:0043549) | 908 | 126 | 82,18 | + | 1,53 | 1,07E-05 | 1,80E-03 |
| positive regulation of cell death (GO:0010942) | 627 | 87 | 56,75 | + | 1,53 | 3,16E-04 | 2,80E-02 |
| response to lipid (GO:0033993) | 822 | 114 | 74,4 | + | 1,53 | 3,56E-05 | 4,88E-03 |
| regulation of cell projection organization (GO:0031344) | 638 | 88 | 57,74 | + | 1,52 | 3,54E-04 | 2,98E-02 |
| animal organ morphogenesis (GO:0009887) | 945 | 129 | 85,53 | + | 1,51 | 1,96E-05 | 2,92E-03 |
| tissue development (GO:0009888) | 1732 | 236 | 156,76 | + | 1,51 | 3,97E-09 | 2,16E-06 |
| negative regulation of cellular protein metabolic process (GO:0032269) | 984 | 134 | 89,06 | + | 1,5 | 1,34E-05 | 2,13E-03 |
